# The Emerging Roles of Long Noncoding RNAs as Hallmarks of Lung Cancer

**DOI:** 10.3389/fonc.2021.761582

**Published:** 2021-10-07

**Authors:** Jun Jiang, Yuan Lu, Fang Zhang, Jie Huang, Xin-ling Ren, Rui Zhang

**Affiliations:** ^1^ Department of Health Service, Fourth Military Medical University, Xi’an, China; ^2^ Department of Pulmonary Medicine, Xijing Hospital, Fourth Military Medical University, Xi’an, China; ^3^ State Key Laboratory of Cancer Biology, Department of Immunology, Fourth Military Medical University, Xi’an, China; ^4^ Department of Respiratory and Critical Care Medicine, Zhongda Hospital, Southeast University, Nanjing, China; ^5^ Department of Pulmonary Medicine, Shenzhen General Hospital, Shenzhen University, Shenzhen, China

**Keywords:** lncRNA, lung cancer, cancer treatment, prognostic biomarkers, predictive biomarkers

## Abstract

Noncoding ribonucleic acids (ncRNAs) are closely associated with tumor initiation, growth, and progress in lung cancer. Long ncRNAs (lncRNAs), as one of the three subclasses of ncRNAs, play important roles in chromatin modification, transcription, and post-transcriptional processing. Various lncRNAs have recently been reported to be dysfunctional or dysregulated in cancers and have pro- or anti-tumor potential. Importantly, as a new class of cancer biomarkers, studies have demonstrated the plausibility of using certain subsets of lncRNAs as promising diagnostic, therapeutic, or prognostic strategies to manage cancers. This review focuses on lncRNAs associated with hallmarks of lung cancer, especially those discovered in the last five years. The expression levels of these lncRNAs in tumor samples are discussed, alongside their mechanisms of action, drug resistance, and potential as diagnostic and prognostic markers for lung cancer.

## Introduction

Lung cancer is one of the leading causes of cancer-related deaths worldwide. Although significantly prolonged survival has been observed in patients undergoing targeted therapies using tyrosine kinase inhibitors (TKIs) and immune checkpoint inhibitors, compared to traditional chemotherapy, the five-year survival rates of lung cancer are still worse in patients with late-stage diseases (varying from 4-17%) (1, 2). This unfavorable outcome results mostly from delayed diagnosis due to a lack of understanding of lung cancer pathogenesis. Identifying reliable predictive biomarkers to screen the disease and access severity is the key to improve the prognosis of lung cancer.

It has been well established that protein-coding genes account for only 2% of the total human genome; approximately 90% of the rest are transcribed as non-coding ribonucleic acids (ncRNAs) (3). NcRNAs can be classified into short ncRNAs and long ncRNAs (lncRNAs) based on length of nucleotides (nt). Short ncRNAs consists of micro RNAs (miRNAs), ribosomal RNA (rRNA), transfer RNA (tRNA), small nuclear RNAs (snRNAs), small nucleolar RNAs (snoRNAs), piwi-interacting RNAs (piRNAs) and other RNAs with known and unknown functions. LncRNAs represent a class of RNA molecules that are typically longer than 200 nt and are less conserved than miRNAs (4). Previous studies have shown that lncRNAs play important roles in chromatin modification, transcription, and post-transcriptional processing through interacting with chromatin-associated proteins (5–7). Most of lncRNAs primarily affect gene expression *in trans* (8), whereas a few, such as X Inactive Specific Transcript (XIST), regulate genomic imprinting *in cis* (9, 10). The basal expression of lncRNAs in human tissues is important for various biological processes, including gene expression, cell differentiation, organogenesis, and homeostasis. Accumulating evidence indicates that their dysregulation in the human genome contributes to the development of human hematologic or solid malignancies, such as XIST (11), HOX antisense intergenic RNA (HOTAIR) (12, 13), and Metastasis Associated Lung Adenocarcinoma Transcript 1 (MALAT1) (14). 78% of the human lncRNAs exhibit more tissue specificity than protein-coding genes, and only ∼19% of the latter have different expression ranges. This means that the functional characterization of tumor-associated lncRNAs (as oncogenes or tumor suppressors) might constitute novel biomarkers for cancers (15, 16).

**Figure 1:**
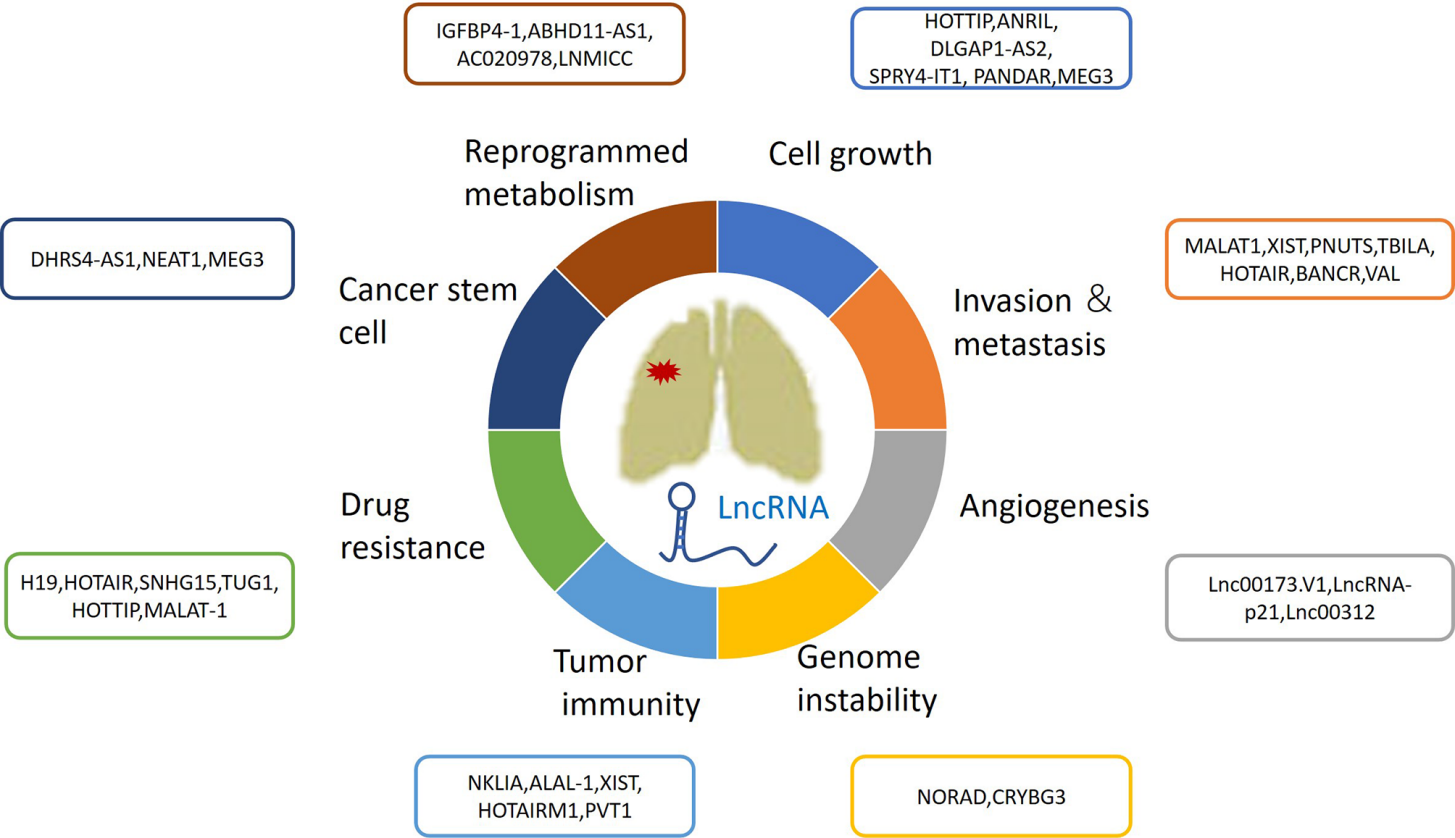
The roles of lncRNAs in lung cancer growth, metastasis, angiogenesis, genomic instability, drug resistance, energy metabolism reprogramming and immune microenvironment and stem cell characteristics.

During the past decade, a growing number of IncRNAs have been found to be aberrantly expressed in lung cancer. Furthermore, increasing attention has been given to lncRNAs as biomarkers for early cancer diagnosis, prognosis, and therapeutic response evaluation. The differential expression of lncRNAs in certain histologic subtypes of lung cancers is associated with diverse tumor prognoses. For example, SRY-Box Transcription Factor 2 (SOX2)-Overlapping Transcript (SOX2-OT) has been frequently detected in lung squamous cell carcinomas (SCCs), rather than in lung adenocarcinomas (LUADs) (17, 18). High expression of SOX2-OT is associated with better outcomes in patients with SCCs, but poor survival in patients with LUADs (19). However, there have been contrasting results regarding the role of SOX2; its association with improved survival might be independent from the histological subtype, emphasizing the need for future thorough studies (20). This review focuses on the roles of lncRNAs in lung cancer biological processes, such as cell growth, metastasis, angiogenesis, genomic instability, drug resistance, energy metabolism reprogramming, immune microenvironments, and stem cell characteristics (**Figure 1** and **Table 1**).

**Table 1:** The lncRNAs associated with lung cancer are listed in alphabetical order.

LncRNA	Intersecting Molecules	Biological Function	Expression	Reference
AGAP2-AS1	silence LATS2 and KLF2 by interacting with EZH2 and LSD1	enhance cell growth	upregulated in NSCLC	(21)
ANRIL	inhibit KLF2 and P21 transcription	promote proliferation, inhibit apoptosis	upregulated in NSCLC	(22)
ALAL-1	induced by TNFα and NF-κB	induce immune evasion	upregulated in NSCLC	(23)
BANCR	Histone deacetylation was involved in the downregulation of BANCR	inhibit metastasis	downregulated in NSCLC	(24)
DHRS4-AS1	sponge miR-224-3p and upregulate tumor suppressors TP53 and TET1	inhibit cancer stem cell	downregulated in NSCLC	(25)
DLGAP1-AS2	increase the expression of cyclin D1	enhance cell growth	upregulated in NSCLC	(26)
H19	upregulation of PKM2	confer erlotinib resistance	upregulated in NSCLC	(27)
HOTAIR	*via* PRC2 (EZH2) and LSD1; interaction with E3 ubiquitin ligases and their corresponding substrates	promote dedifferentiation and proliferation; promote brain metastasis	upregulated in NSCLC and SCLC	(28–30)
HOTTIP	promote BCL-2 by sponging miR-216a	oncogene; promote chemoresistance	upregulated in SCLC	(31, 32)
IGFBP4-1	reprogramed energy metabolism	correlate with TNM stage and lymph node metastasis	upregulated in lung cancer	(33)
Lnc01123	sponge miR-199a-5p to increase c-Myc expression	promote proliferation and aerobic glycolysis	upregulated in NSCLC	(33)
Lnc01537	attenuate Warburg effect and mitochondrial respiration	Inhibit tumor metabolic reprogramming	downregulated in NSCLC	(34)
Lnc00152 Lnc00511	inhibit IL24 and p57 respectively; both interact with EZH2	oncogene	upregulated in NSCLC	(35, 36)
Lnc00673	modulate the expression of a key EMT regulator ZEB1 indirectly	reverse TGF-β induced EMT	upregulated in NSCLC	(37)
Lnc00173	upregulate Etk, GSKIP and NDRG1 by sponging miR-218; translocation of β-catenin	promote chemoresistance and progression	upregulated in SCLC	(38)
Lnc00312	directly binding YBX1	induce migration and vasculogenic mimicry	upregulated in NSCLC	(39)
Lnc01140	sponge miR-377-3 p and miR-155-5 p	promote lung cancer immune escape by elevating PD-L1	upregulated in lung cancer	(40)
LUADT1	suppress p27 by bonding with SUZ12;	promote proliferation	upregulated in LAD	(41)
MEG3	affect p53 expression; modulate PHLPP1 and HIF-1α	inhibit proliferation; pro-apoptosis	downregulated in NSCLC	(42, 43)
MIR22HG	interact with YBX1 and increase its stability; decreased p21 expression	suppress cell proliferation, colony formation, migration, and invasion	downregulated in lung cancer	(44)
MALAT1 (NEAT2)	regulate p53 acetylation; Oct4 activated MALAT1	promote proliferation and motility; affect cell cycle	upregulated in NSCLC	(45–47)
NKILA	Inhibit the activity of NF-κB	induce immune evasion		(48)
NORAD	overactivate of PUMILIO	induce genome instability	upregulated in lung cancer	(49–51)
PANDAR	induce by p53; regulated Bcl-2 *via* interacting with NF-YA	induce cell apoptosis	downregulated in NSCLC	(52)
PVT1	augmented by hypoxia-inducible factor 1 α (HIF1α)	promote the function of MDSCs	upregulated in NSCLC	(53)
PCGEM1	sponge miR-152-3p	promote lymph node metastasis	upregulated in NSCLC	(54)
PNUTS	control the miR-205/ZEB/E-cadherin axis	promote tumor TGFβ-mediated EMT migration	upregulated in NSCLC	(55)
SPRY4-IT1	repress by EZH2	inhibit proliferation, migration and invasion	downregulated in NSCLC	(56)
TARID	activate and demethylate tumor suppressor TCF21	activate tumor suppressor TCF21	silent in NSCLC	(57)
TBILA	enhance RhoA activation *via* cis-regulating HGAL and promote the S100A7- JAB1 pathway by binding to nuclear S100A7 protein	promote tumor TGFβ-mediated EMT migration	upregulated in NSCLC	(58)
TUG1	regulate LIMK2b *via* EZH2 in SCLC; repress HOXB7 *via* EZH2 in NSCLC	chemoresistance to cisplatin	upregulated in SCLC; downregulated in NSCLC	(59)
XIST	regulate miR-367/141-ZEB2 axis; repress KLF2 *via* EZH2	promote tumor TGFβ-mediated EMT migration; induce M2 polarization	upregulated in NSCLC	(60, 61)

## LncRNAs Regulate Lung Cancer Cell Growth

**Figure 2:**
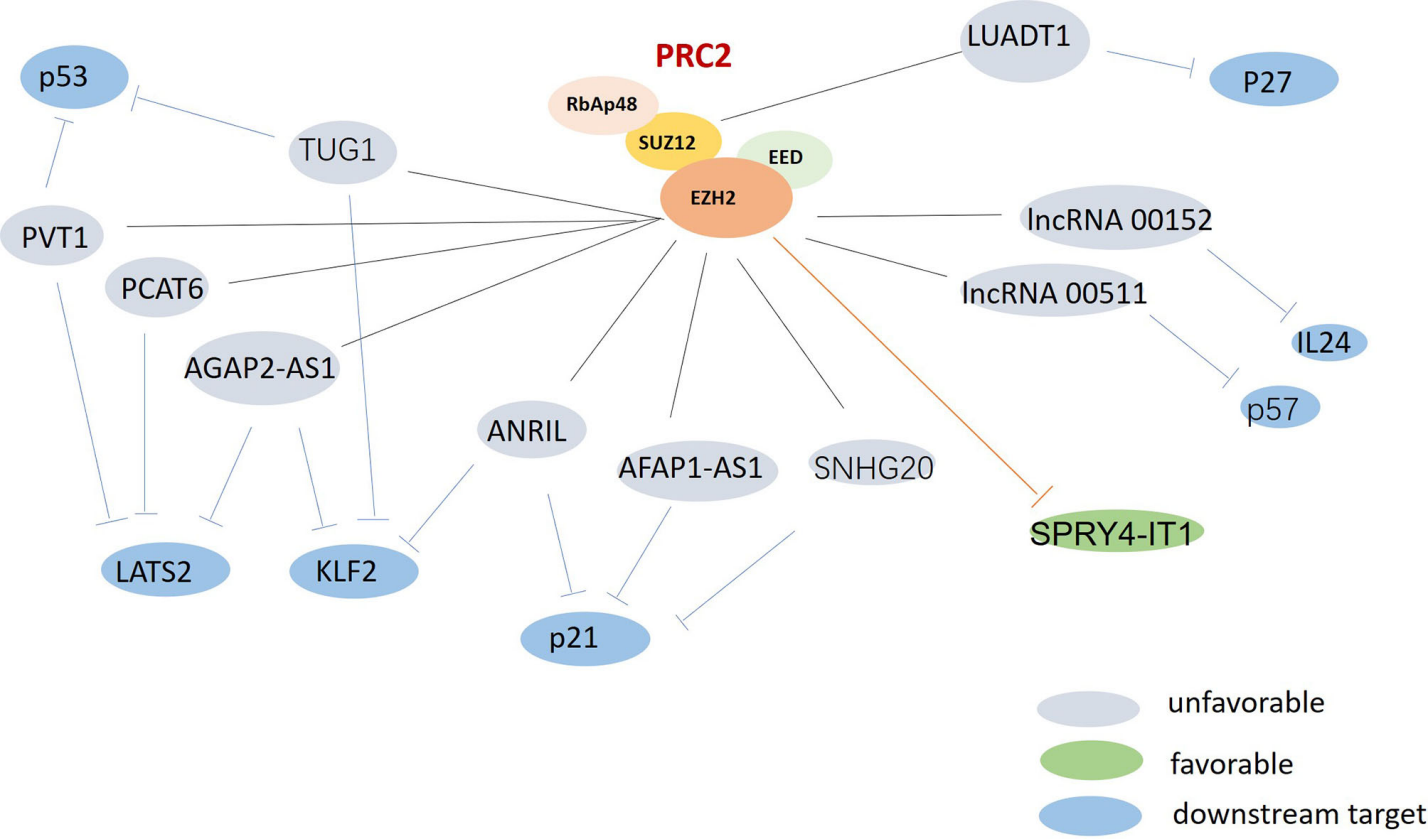
The possible roles of polycomb repressive complex 2 (PRC2) in lung cancer. PRC2 were found upregulated and associated with multi-lncRNAs in lung cancer. Gray: unfavorable lncRNAs. Green: favorable lncRNA. Blue: the downstream targets.

LncRNAs are extensively involved in regulating tumor cell proliferation and apoptosis. However, the specific mechanisms by which lncRNAs affect underlying cancer biological processes remain unclear. Compelling studies into lung cancer have demonstrated that lncRNAs could function as a scaffold for recruiting polycomb repressive complex 2 (PRC2) or a methyltransferase for histone H3 lysine 27 trimethylation (H3K27me3). They could also consist of enhancer of zeste homolog 2 (EZH2), be a suppressor of zeste 12 (SUZ12), could influence embryonic ectoderm development (EED) and retinoblastoma associated protein 48 (RbAp48), or could act as either oncogenes or tumor suppressors, depending on the effectors interacted (**Figure 2**).

### LncRNAs Promote Lung Cancer Cell Growth

EZH2, which is the catalytic subunit of PRC2, is often over-expressed in lung cancer (62). LncRNAs can recruit EZH2 to the promoter region of certain genes, and can regulate their expression. EZH2-mediated H3K27 methylation can also silence many tumor suppressors, leading to tumorigenesis and poor prognoses. Recent studies have revealed that the oncogenic lncRNAs AGAP2 Antisense RNA 1 (AGAP2-AS1) and ANRIL can promote tumor cell growth by repressing the expression of the suppressors Large Tumor Suppressor Kinase 2 (LATS2) (21), Kruppel Like Factor 2 (KLF2) (22), and p21 (63) *via* interacting with EZH2 in non-small cell lung cancer (NSCLC). HOXA Distal Transcript Antisense RNA (HOTTIP) has been identified as upregulated lncRNA in small cell lung cancer (SCLC) tissues, and to be correlated with shorter survival times for SCLC patients. HOTTIP knockdown can impair cell proliferation and inhibit tumor growth by sponging miR-574-5p and repressing the expression of EZH1 (31). A better understanding of lncRNA-PRC2 interactions could provide new insights for the development of lung cancer treatments.

Cyclin-dependent kinases (CDKs) and CDK inhibitors (CKIs) play vital regulating roles in cell cycle, which is universally altered in cancer. The lncRNAs lnc00152 and lnc00511, which have been found to be upregulated in LUAD tissues, can play oncogenic roles by potently inhibiting the tumor suppressor IL24 (35) and the CKI p57 (36), respectively. Another vastly assessed oncogenic lncRNA is lung adenocarcinoma transcript 1 (LUADT1). LUADT1 has been shown to be highly expressed in LUAD, and to be correlated with histological T staging; it can also promote cancer cell proliferation upon binding with SUZ12 and mediate the trimethylation of H3K27 at the promoter region of the tumor-suppressor p27; this was found to epigenetically downregulate the expression of P27 (41). Although many lncRNAs have been reported to promote the proliferation of lung cancer, Wang Lu recently performed bioinformatics analysis and found DLGAP1-AS2 as another new lncRNA in NSCLC led to sustained tumor cell proliferation. The function of DLGAP1-AS2 has only been analyzed in glioma, but its role in other tumors is still unknown. Wang Lu found DLGAP1-AS2 was upregulated in NSCLC and correlated with the poor survival of NSCLC patients. DLGAP1-AS2 could promote NSCLC cell proliferation by increasing the expression of cyclin D1, a target of miR-503 (26).

### LncRNAs Inhibit Lung Cancer Cell Growth

**Figure 3:**
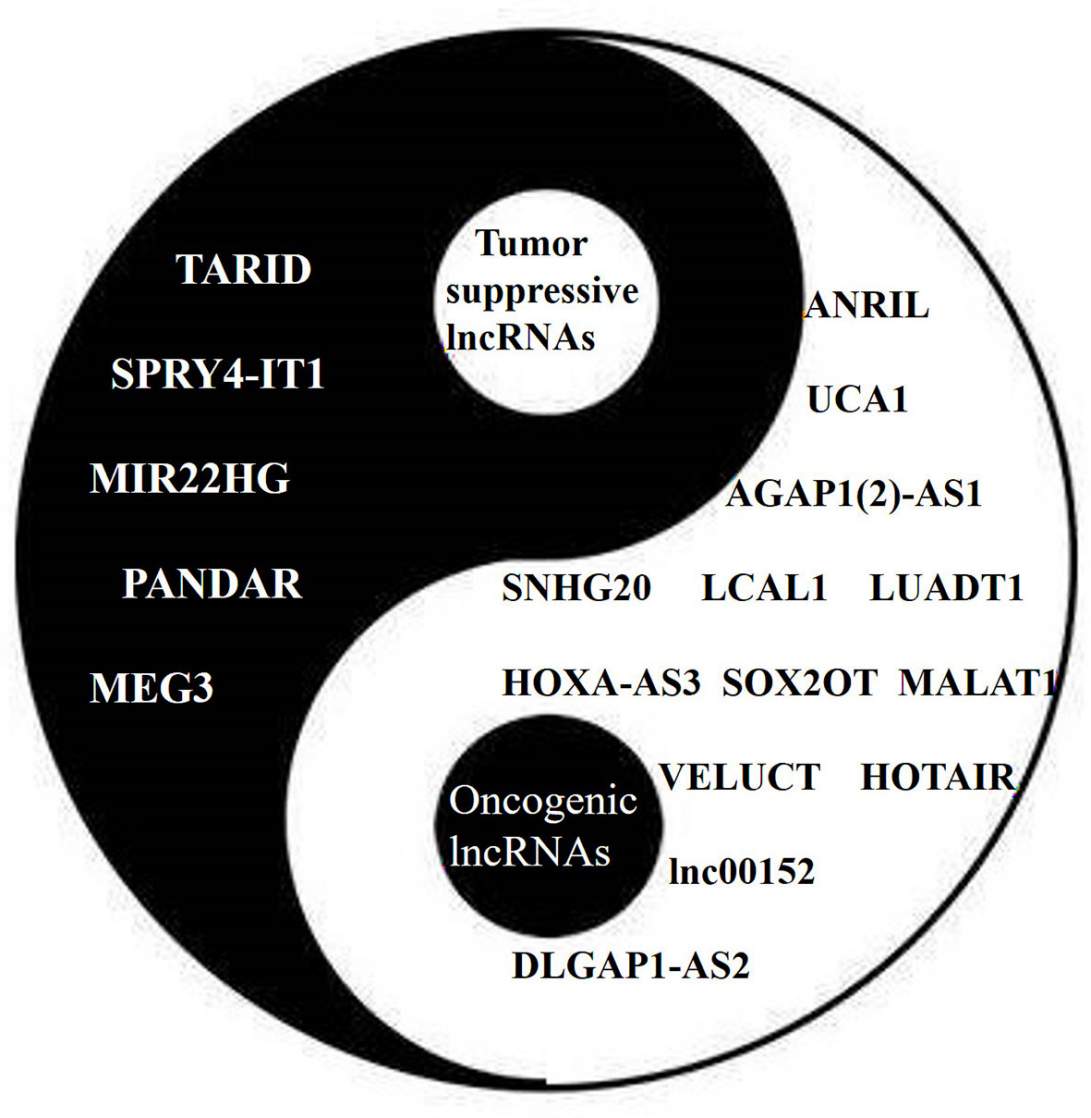
The Yin (tumor suppressive) and Yang (oncogenic) of lncRNAs in lung cancer. Different lncRNAs play different roles in lung cancer similar to Yin and Yang.

Some lncRNAs function as tumor suppressors that are usually downregulated in lung cancer (**Figure 3**). The expression of the lncRNA SPRY4 Intronic Transcript 1 (SPRY4-IT1) has been found to be repressed in NSCLC by EZH2. The transfection of SPRY4-IT1 into NSCLC cells has been shown to result in a significant antitumoral effect, both *in vitro* and *in vivo* (56). Maternally Expressed 3 (MEG3) has also been found to be downregulated in NSCLC, while exogenous MEG3 has been shown to significantly inhibit cell proliferation and induce cell apoptosis through the activation of p53 (42). The expression of MIR22 Host Gene (MIR22HG) has also been shown to be significantly decreased in lung cancer tissues, and to be associated with poor patient outcomes. An *in vitro* study suggested that MIR22HG can prevent Y-Box Binding Protein 1 (YBX1) from improving the expression of MET, and can abolish the oncogenic function of p21 in the cell cycle (44).

Furthermore, LncRNAs have been reported to participate in deoxyribose nucleic acid (DNA) methylation machinery to mediate chromatin-based gene regulation. Transcription factor 21 (TCF21) is a well-characterized tumor suppressor. CGI1 and CGI3, two exons that encode for TCF21, have been shown to be unmethylated in normal lung tissue samples and to be hypermethylated in NSCLC samples. The lncRNA TCF21 Antisense RNA Inducing Promoter Demethylation (TARID) has been shown to mediate the expression, demethylation, and activation of TCF21 when interacting with the DNA demethylation regulators GADD45A, TDG, and TET. Silencing TARID in cancer cells was found to result in the aberrant methylation of CGI1 and CGI3 (57). LncRNAs have also been shown to be involved in the inhibition of tumor cell proliferation in lung cancer through a P53-associated pathway. For example, P53 can suppress tumorigenesis by inhibiting PVT1b (the isoform of PVT1)-dependent Myc transcription (64). The lncRNA Promoter of CDKN1A Antisense DNA Damage Activated RNA (PANDAR) is a direct transcriptional target of p53; it has generally been found to be downregulated in NSCLC tissues. A study showed that the cell viabilities of lung cancer cell lines could be significantly abrogated by the overexpression of PANDAR, which was shown to detain and disable the Bcl-2 transcription factor Nuclear Transcription Factor Y Subunit Alpha (NF-YA) (52). In general, lncRNAs are important components of the p53 regulatory network. Further studies into characterizing the p53-lncRNA relationship will not only facilitate the understanding of lncRNA-mediated gene regulation, but will also provide potential therapeutic targets in lung cancer.

## LncRNAs in Lung Cancer Metastasis

Metastasis is the major cause of death in lung cancer. The epithelial-mesenchymal transition (EMT) process is considered as the key factor in malignant stemness and is the first critical step of cancer cell metastasis. It is, therefore, of great significance to explore new biomarkers to diagnose lung cancer at an early stage. Metastasis Associated Lung Adenocarcinoma Transcript 1 (MALAT-1) is the most well-characterized lncRNA linked to lung cancer metastasis; it regulates cancer development by functioning as a competing endogenous RNA (ceRNA) to sponge a series of miRNAs. For example, miR-145 has been reported to decrease the levels of Smad3 messenger RNA (mRNA) by interacting with its three prime untranslated region (3′UTR), whereby EMT and invasive processes cease in A549 cells (65). Conversely, elevated levels of MALAT-1 could reduce the expression of miR-145, which would allow tumor cells to avoid the cell death induced by chemotherapy (45).

Transforming growth factor-β (TGF-β) is known to induce EMT and regulate lung cancer metastasis. Several lncRNAs have been shown to promote TGF-β-induced EMT signaling in lung cancer cells. XIST, which has been shown to be upregulated in various types of solid tumors, as well as in NSCLC, could promote TGF-β-induced EMT by sponging miR-367/141 to enhance the expression of the transcription factor Zinc finger E-box-binding homeobox 2 (ZEB2) in NSCLC (60). In A549 and NMuMG cells, the lncRNA phosphatase 1 nuclear targeting subunit (PNUTS) has been shown to promote TGFβ-mediated EMT, and to enhance tumor cell migration and invasion by controlling the miR-205/ZEB/E-cadherin axis (55). Lnc00673 has also been found to sponge miR-150-5p, and to indirectly modulate the expression of the EMT-regulator ZEB1. The knockdown of lnc00673 has been found to reverse TGF-β-induced EMT in *in vitro* experiments, and to attenuate the tumorigenesis ability of A549 cells *in vivo* (37). The upregulation of TGF-Beta Induced LncRNA (TBILA) has also been shown to promote NSCLC cell migration and invasion by inducing EMT, through the enhancement of HGAL expression, and through the activation of Ras Homolog Family Member A (RhoA) (58). EMT has been shown to be accompanied by focal DNA hypermethylation. A high throughput study has shown that the potential of lncRNA Hox transcript antisenses RNA (HOTAIR) in prediction of poor prognoses for lung cancer patients. An *in vitro* study demonstrated that HOTAIR NSCLC transfectants showed abnormal cell cycles, due to the mediation of EMT by PRC2-catalyzed H3 lysine 27 methylation (28, 29, 66). H3K27 methyltransferase EZH2 is the enzymatic subunit of PRC2. Inhibiting the expression of EZH2 could significantly block EMT. The enhanced interaction between EZH2 and Lnc01234 has been shown to promote NSCLC metastasis *via* histone modification, and *via* the transcriptional repression of the anti-proliferative gene B-cell translocation gene 2 (BTG2) (67).

Metastasis may also occur in the absence of EMT. The overexpression of the lncRNA vimentin associated lncRNA (VAL) was shown to strikingly promote local invasion and distant metastasis in LUAD cells through AKT signaling, without inducing phenotypic or molecular changes in the EMT. Additionally, silencing VAL could attenuate bone lesions and improve distant metastasis-free survival (68). A few lncRNAs have been shown to have inhibitory effects on EMT in lung cancer. For example, the expression of lncRNA BRAF-Activated Non-Protein Coding RNA (BANCR) significantly decreases in NSCLC tumor tissues, and the ectopic expression of BANCR inhibits the metastatic abilities of SPC-A1 and A549 cells (24). Another lncRNA is prostate cancer gene expression marker 1 (PCGEM1), Huang J reported, elevated in NSCLC. PCGEM1 can promote the occurrence and progression of prostate cancer, but its role in lung cancer is still unclear. Huang’s research team recently discovered that PCGEM1 contributed to lymph node metastasis and deterioration in NSCLC patients. Additionally, knockdown of PCGEM1 inhibits NSCLC cell invasion and migration by sponging miR-152-3p, indicating PCGEM1/miR-152-3p axis may be novel therapeutic targets for NSCLC treatment (54).

## LncRNAs in Lung Cancer Angiogenesis

The induction of tumor angiogenesis, which is a pathologic process that is critical for tumor growth, invasion, and metastasis, is mediated by various angiogenic factors and complex signaling pathways major including VEGF, Notch and PI3K/AKT signaling pathways. Vascular endothelial growth factor (VEGF) is the primary mediator of the proliferation and migration of vascular endothelial cells. The anti-VEGF antibodies Bevacizumab and Ramucirumab have been recommended for treating patients with advanced NSCLC (69, 70). The lncRNAs involved in VEGF signaling may also be ideal targets for inhibiting lung cancer angiogenesis. Lnc00173.v1 could promote the migration of vascular endothelial cells by upregulating the expression of VEGFA *via* sponging miR-511-5p in lung squamous cell carcinoma (LSCC) (38). Hypoxic conditions are essential for tumor angiogenesis. A previous study showed that the overexpression of lncRNA-p21 in NSCLC under hypoxic conditions produced a strong pro-angiogenetic effect— lncRNA-p21 was found to significantly enhance tube formation in endothelial cells and stimulate tumor cell adhesion. Additionally, lncRNA-p21 silencing has been found to reduce the expression of VEGFA and other pro-angiogenic factors, such as Matrix Metallopeptidase 2 (MMP2), Platelet Derived Growth Factor Subunit B (PDGFB), and Fibroblast Growth Factor 2 (FGF2). Moreover, lncRNA-p21 levels in blood have been shown to be an independent prognostic marker for the time to relapse and the overall survival (71).

Vasculogenic mimicry (VM) means that tumor cells do not depend on endothelial cells, but on adjacent tissue to supply nutrition (72). Tumor cells provide blood supply by mimicking the formation of blood vessel wall structure through self-deformation and extracellular matrix interaction. VM has been observed in various types of malignancies, including lung cancer. As a promising target for anti-cancer therapy, VM is essential for lung cancer growth and metastasis (73). Lnc00312 has been shown to induce VM in lung adenocarcinoma, and to potentiate cell migration by directly binding to YBX1 (39). The expression of MALAT1, which is upregulated by the binding of estrogen receptor β (ERβ) on its promoter, could promote VM by reversing the miR145-5p-mediated downregulation of Neural Precursor Cell Expressed, Developmentally Down-Regulated 9 (NEDD9) (46). Furthermore, the survival of ERβ-positive NSCLC female patients has been shown to be worse than that of ERβ-negative NSCLC female patients. The combination of anti-VEGR and anti-VM medicines might be useful in treating lung cancer.

## LncRNAs Mediate Drug Resistance of Lung Cancer

Lung cancers are composed of different subgroups of cancer cells with different cancerous phenotype expression and drug sensitivities (74). LncRNAs are also involved in the multi-drug resistance of lung cancer, including TKI resistance and chemoresistance.

### LncRNAs Regulate TKI Resistance

In the last decades, TKIs have been widely used in NSCLC patients harboring active mutations. However, the efficacy of this approach is hampered by primary and acquired resistance. Recent studies have shown that lncRNAs play important roles in the resistance to TKI targeting Epidermal Growth Factor Receptor (EGFR) and ALK signaling.

The lncRNAs H19 and HOTAIR have been found to be highly expressed in EGFR-TKI-sensitive lung cancer tissues, but not expressed in resistant samples. H19 has been found to be downregulated in cases of NSCLC harboring the T790M mutation after TKI treatment, compared to pretreatment tissues. A study found that knocking down H19 *in vitro* conferred Erlotinib resistance through the upregulation of PKM2 and the phosphorylation of AKT (27). HOTAIR has been shown to be suppressed in EGFR-TKI-resistant lung cancer cell lines and tissues, while HOTAIR transfectants restored sensitivity to EGFR-TKIs (30). Long Intergenic Non-Protein Coding RNA 665 (LINC00665) could also induce TKI resistance and promote the invasive ability of lung cancer cells by interacting with EZH2 and activating the AKT pathway. Silencing Lnc00665 was shown to result in fetal growth restriction and apoptosis in lung cancer cell lines treated with Gefitinib (75). Cytoplasmic Small Nucleolar RNA Host Gene 15 (SNHG15) has been demonstrated to potentiate the expression of multidrug resistance protein 1 (MDR-1) in GR cancer cells by sponging the tumor suppressor miR-451 *via* the NOTCH signaling pathway (76).

The transfer of extracellular vesicles (EVs) that are derived from ALK TKI-resistant subclones to ALK TKI-sensitive subclones has been shown to cause drug-resistance in the originally sensitive ones. EV-RNA profiling has revealed that MEG3 and XIST may be associated with ALK-TKI resistance, which have been shown to be highly expressed in EVs secreted by ALK-TKI resistant subclones (77).

### LncRNAs Mediate Chemoresistance

Chemotherapy is the first-line treatment for advanced lung cancer. However, chemoresistance remains a major obstacle to successful treatment. Recent studies have shown that several lncRNAs are associated with cisplatin (DDP) and docetaxel (DTX) resistance. The lncRNA SOX2-OT is involved in cisplatin resistance and has been shown to be correlated with poor clinical prognosis. This may in part be due to the activation of AKT signaling and increased expression of SOX2 and GLI-1 (78). SOX2-OT silencing has been shown to sensitize lung cancer cells to cisplatin. Inhibiting MALAT1 transcription, which is regulated by Transcription factor AP-2 gamma (TFAP2C) and ZEB1, has been shown to release miR-200b and restore the sensitivity of LUAD cells to DTX treatment (47).

SCLC is an aggressive disease in which patients rapidly relapse after an initial response to chemotherapy. LncRNA plays an important role in SCLC biology; it might be helpful in the development of therapeutic strategies for SCLC patients. The upregulation of Taurine Up-Regulated 1 (TUG1) increases the expression of LIMK2b (LIM kinase family) upon binding with EZH2, thereby conferring DDP resistance to SCLC cells. Furthermore, TUG1 knockdown significantly enhances the chemosensitivity of drug-resistant H69 and H446 cells, both *in vitro* and *in vivo* (59). The lncRNA HOTTIP frequently amplifies in SCLC and contributes to multidrug chemoresistance, such as Adriamycin, cisplatin, and VP-16, by promoting the expression of BCL-2 through sponging miR-216a (32). Lnc00173 could mediate chemoresistance in SCLC by upregulating GSK3β interacting protein (GSKIP), and N-Myc Downstream Regulated 1 (NDRG1) *via* sponging miR-218 (79).

## LncRNAs in Lung Cancer Genome Instability

As one of the hallmarks of cancer, genomic instability participates in different cell cycle checkpoints after DNA damage. This can be observed in a variety of malignant tumors and precancerous lesions. Genomic instability can be manifested as entire chromosome gain or loss (aneuploidy), chromosomal translocation (fusion gene), rearrangement, or telomere structure changes (80).

The lncRNA non-coding RNA activated by DNA damage (NORAD, lnc00657) is highly conservative and is abundantly expressed in many types of cells; it can assemble a topoisomerase complex, which is essential for genome stability (49). NORAD has been shown to sequester PUMILIO to maintain genomic stability, and the loss of NORAD has been demonstrated to result in the overactivation of PUMILIO. It has been shown that cells with overactivated PUMILIO cannot transfer chromosomes correctly during mitosis (50). NORAD has also been found to be upregulated in NSCLC cells undergoing DNA replication stress and DNA damage. For example, NORAD has been shown to be upregulated in A549 lung cancer cells in response to exposure to airborne particulate matter <10 µm (PM_10_). This induced an increase in micronuclei, which are a phenotype of chromosomal instability (51). Crystallin Beta-Gamma Domain Containing 3 (CRYBG3) is a another upregulated lncRNA resulting in genomic instability in A549 lung cancer cells. The overexpressed of CRYBG3 has been shown to interfere with spindle assembly checkpoint activities through inhibiting the interaction between Bub3 and CDC20, resulting in mitotic catastrophes and the formation of aneuploidy; this can eventually promote tumorigenesis and metastasis in NSCLC (81).

The genomic instability of SCLC is characterized by high mutation burdens, the defection of the tumor suppressor TP53, and the amplification of Myc (82). Cyclin-dependent kinase 7 (CDK7) is a protein kinase that influences cell division and gene transcription. The inhibition of CDK7 using the selective inhibitor YKL-5-124 has been shown to have a synthetic lethal effect when combined with a TP53 activator; this can disrupt cell cycle progression, induce DNA replication stress, and trigger genome instability in SCLC (83). Importantly, genome instability and the formation of micronuclei induced by YKL-5-124 have been shown to trigger T-cell dominated antitumor immunity in SCLC. The combination of YKL-5-124 and an immune-checkpoint blockade can effectively inhibit the proliferation of SCLC; this provides a theoretical basis for a promising combination therapy consisting of CDK7 inhibition and anti-PD-1 antibody application in SCLC treatment (84).

## LncRNAs in Lung Cancer Immune Evasion

Suppressing the immune system is vital for the survival and development of tumor malignancy. Tumor cells can escape from immune surveillance and destruction by forming a tumor-promoting microenvironment. Tumor cells can secrete a wide range of substances to inhibit the cytotoxic functions of tumor-antagonizing immune responses, including cytokines and chemokines. LncRNA plays a highly prominent role in regulating the expression and function of these cytokines and chemokines during the development and differentiation of immune cells (85). Interferon gamma (IFN-γ), when released by T cells, can facilitate the transcription of the lncRNA NF-KappaB Interacting LncRNA (NKILA) through Janus kinase-signal transducer and activator of transcription (JAK-STAT1) signaling. This consequently tips the immunoactive and immunosuppressive balance in a tumor microenvironment (TME). Upon interacting with NF-κB, the overexpression of NKILA can induce the apoptosis of tumor-specific cytotoxic T lymphocytes (CTLs) and type 1 helper T cells (TH1) in lung cancer. Importantly, higher levels of NKILA have been suggested to be related to shorter disease-free survival in NSCLC. Conversely, STAT1 inhibition has been shown to be efficiently abolished through NKILA upregulation, and to prevent the cell apoptosis of CTLs and TH1 in TME (48). The upregulation of the lncRNA ALAL-1, which is another transcriptional target of NF-κB, has been shown to be inversely associated with the immune infiltration of T cells in NSCLC. Tumors with higher levels of ALAL-1 have been shown to express lower levels of PD-1 and lesser immune populations, such as T memory, T follicular helper, and dendritic cells. This resulted in the production of pro-inflammatory mediators such as CXCL1, IL-6, and CXCL10, which would otherwise attract immune cells to kill cancer cells, being excluded from TME (23). Moreover, lnc01140 is highly expressed in a variety of tumors including lung cancer, but its biological role in lung cancer immune escape is still unknown. Xia Rongmu found that high lnc01140 level promoted lung cancer immune escape and correlated with poor survival in patients. Lnc01140 elevated programmed death-ligand 1 (PD-L1) and c-Myc expression by repressing multiple microRNAs including miR-377-3 p and miR-155-5 p. *In vivo* assay, downregulation of Lnc01140 repressed the growth of subcutaneous lung cancer xenografts by inhibition of PD-L1 expression (40).

## LncRNAs in Lung Cancer Associated Inflammation

The participation of inflammatory cells and their products in the formation of TME has attracted enormous interest. In pulmonary scar cancer, chronic inflammation can promote tumorigenesis long before malignant changes occur. However, tumorigenic changes are not always preceded by long-standing chronic inflammation, which could also be elicited by tumor cells, and could contribute to the formation and reshaping of TME, which in turn can benefit tumor development. Upon binding to corresponding receptors, chemokines promote tumor angiogenesis, tumor cell migration, and the escape of anti-tumor immune surveillance (86, 87). Pro-inflammatory mediators, including NF-κB, the STAT3 transcription factor, and ILs cytokines are closely related to neoplastic disorders; they contribute greatly to shaping TME.

Macrophages can be divided into two phenotypes, M1 and M2. Type 1 macrophages (M1) are potent effector cells, which are characterized by the secretion of IL-12 and inducible nitric oxide synthase (iNOS); they kill microorganisms and tumor cells. Type 2 macrophages (M2), meanwhile, can polarize macrophages that express high levels of IL-10 and Arg-1, which facilitates the remodeling of blood vessels and the inhibition of adaptive antitumor immunity to predispose tumor angiogenesis and metastasis (86). Tumor-associated macrophages (TAMs) are key components of leukocyte infiltration and inflammatory circuits in TME, as they exhibit the properties of a polarized M2 population (88). LncRNAs can regulate the inflammatory response by modulating the transcriptional control of inflammatory cells, including TAMs. The upregulation of the lncRNA XIST has been shown to promote M2-like TAM polarization by directly binding to Transcription Factor 4 (TCF-4). TCF-4 functions as a nuclear response; it can affect cell proliferation, differentiation, and apoptosis *via* Wnt signaling in TAM (61).

Myeloid-derived suppressor cells (MDSCs) and dendritic cells (DCs) also respond and adapt to the inflammatory signals in TME during tumorigenesis. MDSCs are a heterogeneous population of immature myeloid cells with immunosuppressive characteristics. HOXA transcript antisense RNA myeloid-specific 1 (HOTAIRM1) has been shown to be significantly underexpressed in MDSCs in lung adenocarcinoma tissues, compared to adjacent normal tissues. HOTAIRM1 has been found to enhance the expression of HOXA1 in MDSCs, and the upregulation of HOXA1 can delay tumor progression and enhance anti-tumor immune response by restraining the immunosuppressive activity of MDSCs. Moreover, patients with lung cancer have exhibited lower levels of peripheral blood HOTAIRM1 than healthy controls, indicating that HOTAIRM1 might be an ideal biomarker for diagnosing lung cancer (89). The lncRNA AK036396 has also been shown to potentiate the immunosuppressive ability of MDSCs by enhancing the stability and expression of Ficolin B, which is an ortholog of human M-Fcnb, in the MDSCs of tumor tissues isolated from a mouse model (90).

LncRNAs also play significant anti-tumorigenic roles by reversing immunosuppression of MDSCs in lung cancer. In response to hypoxia, the expression of the lncRNA PVT1 in MDSCs can be augmented by hypoxia-inducible factor 1 α (HIF1α). Notably, PVT1 knockdown has been shown to result in the inhibited immunosuppressive function of MDSCs *in vitro*, and to improved pro-tumor activity *in vivo* (53). When highly expressed, lnc00301 functions as an oncogenic regulator in lung cancer. Lnc00301 has been shown to accumulate regulatory T cells (Treg), but can repress CTL infiltration in the TME of tumors isolated from the xenograft mice model by binding directly to the enhancer of EZH2 to downregulate ELL Associated Factor 2 (EAF2) (91).

## LncRNAs in Lung Cancer Metabolism Reprogramming

Cancers cells can efficiently alter metabolic pathways to facilitate nutrient uptake and incorporation into cells, which are in a highly proliferating status. Various transcription factors and metabolic enzymes are involved in cancer metabolic alterations, and some have been documented as regulatory targets of lncRNA. Understanding of the roles of lncRNAs in metabolism reprogramming may help to provide novel theranostic markers for lung cancer.

The Warburg effect means that cancer cells activate glycolysis for adenosine tri-phosphate (ATP) generation to sustain a high rate of cell proliferation and growth, even under aerobic conditions; this is also known as aerobic glycolysis (92). LncRNAs can directly regulate the expression of glycolytic enzymes and glucose transporter (GLUT), or can affect the signal transduction pathways that control energy metabolism (93). The upregulation of the lncRNA ABHD11-AS1 in NSCLC due to the Methyltransferase-like 3 (METTL3)-mediated m^6^A modification could promote the Warburg effect and enhance cell proliferation (94). The overexpression of the lncRNA IGFBP4-1 in lung cancer has been demonstrated to play a positive role in cell proliferation and metastasis; this could reprogram tumor cell energy metabolism, which could feature the increased production of ATP and the upregulated expression of metabolic enzymes, including HK2, PDK1, and LDHA (33). Lnc01123 has been shown to promote NSCLC cell proliferation and aerobic glycolysis by sponging miR-199a-5p to increase c-Myc expression (95). Under hypoxia, the lncRNA AC020978 has been found to promote cell proliferation and the glycolytic metabolism of NSCLC, through the PKM2/HIF-1α axis (96). Nevertheless, there are lncRNAs that exert protective effects against lung cancer as well. Lnc01537 has been shown to stabilize PDE2A mRNA to attenuate the Warburg effect, and mitochondrial respiration (34).

Fatty acids are important energy resources for cancer cell growth and survival. In addition to being the main component of membrane matrix structures, the products of fatty acid metabolism also play important roles in carcinogenic signaling transductions and cell membrane homeostasis as important secondary messengers or energy sources. Cancer cells mainly rely on endogenous *de novo* synthesis to produce fatty acids, which consumes significant amounts of ATP and Nicotinamide adenine dinucleotide phosphate (NADPH), instead of ingesting exogenous fatty acids (97). LncRNAs have been well documented to have a potent influence upon fatty acid production in cervical and gastric cancers. The lncRNA LNMICC has been shown to promote the nodal metastasis of cervical cancer by reprogramming fatty acid metabolism. Furthermore, silencing LNMICC has been shown to significantly decrease the levels of intracellular triglycerides and phospholipids by influencing the expression of the metabolic enzymes of fatty acids (98). In gastric cancer, the lncRNA MACC1-AS1 has been found to promote stemness and chemoresistance by maintaining low cellular reactive oxygen species (ROS) by activating the oxidation of fatty acids (99). However, lncRNAs tend to be expressed in a spatio-temporal and cell type specific manner. The roles of lncRNAs in the lipid metabolism of lung cancer cells have rarely been reported. Future studies should pay more attention to identifying lncRNA that are related to fatty acid metabolism, in the context of lung cancer.

## LncRNAs in Lung Cancer Stem Cells (CSCs)

CSCs endow lung cancer cells with the capacity for limitless self-renewal; they also play vital roles in the multidrug resistance of lung cancer. The phenotypes of CSCs are characterized by asymmetric cell division, drug resistance, and the ability to trigger distant metastasis. Accumulating evidence indicates that lncRNAs play important roles in maintaining the stemness properties and tumorigenicity of CSCs in NSCLC (100). Achieving a better understanding of lncRNAs in the properties of CSCs might facilitate the development of lung cancer therapies.

CSC populations commonly express a number of cell surface markers, such as sex determining region of Y related high mobility group box2 (Sox2), octamer-binding transcription factor 4 (Oct4), Nanog, aldehyde dehydrogenase (ALDH), CD34, CD44, CD133, and CD166 (101). Squamous cell carcinoma (SCC) and adenocarcinoma have distinct expression profiles in CSC markers. CD44, Sox2, and ALDH1 have frequently been found in CSCs from SCC, whereas CD166 has been found to be enriched in CSCs from adenocarcinomas (102). Importantly, Sox2 and Oct4 are transcription factors that are required to maintain the self-renewal properties of NSCLC (103). The overexpression of the lncRNA lnc00662, which is a newly identified lncRNA related to human lung cancer, has been shown to enhance H1299 and A549 lung cancer cell metastasis and CSC stemness by directly interacting with the pro-tumor protein Lin28 (104). In NSCLC stem cells, the elevated expression of the lncRNA Nuclear Paraspeckle Assembly Transcript 1 (NEAT1) has been shown to suppress the transcription factor CTR1 and improve the expression of Sox2. Furthermore, Oct4 has been shown to induce CSC-like phenotypes *via* the Wnt/β-catenin and EMT pathways (105). Furthermore, in addition to inducing a CSC-like state, some lncRNAs are well documented to be protective factors against neoplastic expansion. The lncRNA MEG3 has been shown to successfully alleviate CSC-like states and the metastasis of H1299 cells. Silencing MEG3 in H1299 cells has been shown to release miR-650 (which targets SLC34A2), resulting in the upregulation of Oct4 and CD133 (43). The overexpression of the lncRNA DHRS4-AS1 is related to the inhibition of cancer stemness in NSCLC *via* abrogation of the expression of Sox2, Oct4, CD34, and CD133 through sponging of miR-224-3p and upregulation of the tumor suppressors TP53 and TET1 (25, 101, 106). In summary, lncRNAs have specific expression profile in tumor stem cells, which means lncRNAs might be a potential approach to killing CSCs and preventing tumor recurrence. Moreover, lncRNAs could be promising biomarkers to predict the recurrence probability according to specific expression in plasma released by hidden CSCs.

## Conclusions and Expectations

The dysregulated expression of lncRNAs can cause cellular imbalance and result in cancerogenesis, making lncRNAs as potential diagnostic and prognostic markers for lung cancer. With advances in RNA-sequencing techniques, increased numbers of lncRNAs have been identified in lung cancer. However, up to now only a few lncRNAs have been experimentally studied and well-described in lung cancer biology. In this review, we summarized the regulatory role of lncRNAs in lung cancer from the perspectives of hallmarks of cancer (*i.e.* cellular proliferation, metastasis, angiogenesis, drug resistance, stem cell characteristics, genome instability, immune escape, and energy metabolism reprogrammed of lung cancer), aiming at providing broad and effective strategies for lung cancer treatments.

Developing technologies and tools to target lncRNAs and lncRNA-based drugs have been widely applied in lung cancer treatment. Strategies employing gene silencing technology have been proposed to upregulate the tumor suppressive effects of lncRNAs, including Antisense oligonucleotides (ASOs), RNA interference (RNAi), and CRISPR/Cas9 (107). ASOs can attenuate the pro-tumor functions of lncRNA strands by directly binding to them and exhibit a synergistic anti-tumor effect in clinical studies when combined with RNAi technology (108), chemotherapeutic drugs (38), small molecule inhibitors (109), immune checkpoint antibodies (110), etc., which means lncRNA could be the appropriate targets for lung cancer treatment.

However, the modes of action of lncRNAs are complicated somewhat by the fact that multiple interactions with chromatin, protein, and RNA are involved, and that complex regulatory networks are composed. Therefore, although they have been studied for the last decade, the detailed molecular mechanisms of lncRNAs in lung cancer are still insufficiently explored. For future expectations, more novel lncRNAs could be discovered by the established on-line bioinformatic algorithms to provide comprehensive annotations about their secondary structures, identify interactions with other RNAs and proteins, and evaluate tissue-specificities and subcellular localizations, and eventually strengthen our understanding of regulation pathway among lncRNAs and find more potential therapeutic targets. On the other hand, gene therapy targeting lncRNA is an emerging strategy, but off-target effects can easily lead to adverse effects. The nano drug delivery system can transport siRNA to target lncRNA of tumor tissues with high specificity. Studies have demonstrated that ASOs conjugated with nanoparticles can exhibit great potential for the treatment of lung cancer metastasis (111). Additionally, the improved chemical modifications of ASOs have also been employed to address off-target effects (112). Given that lncRNAs are dysregulated in lung cancer, it shows great potential to suppressing the initiation and progression of lung cancer *via* manipulating their expression levels. Nevertheless, the challenge nowadays is to transfer these findings and research to the clinical application and to improve the early detection and prognostication for the patients with lung cancer, and it also needs further *in vivo* and *in vitro* studies and clinical investigation to explore and verify the effectiveness and safety of lncRNA drugs.

## Author Contributions

All authors participated in the discussion of the draft. JJ and YL: Writing—original draft. FZ and JH: Validation and writing—revision and editing. X-lR: Supervision. RZ: Conceptualization, supervision, and project administration. All authors contributed to the article and approved the submitted version.

## Funding

This work was supported by the National Natural Science Foundation of China (81871880, 81773262) and Jiangsu Basic Research Program (Natural Science Foundation, No. BK20201484).

## Conflict of Interest

The authors declare that the research was conducted in the absence of any commercial or financial relationships that could be construed as a potential conflict of interest.

## Publisher’s Note

All claims expressed in this article are solely those of the authors and do not necessarily represent those of their affiliated organizations, or those of the publisher, the editors and the reviewers. Any product that may be evaluated in this article, or claim that may be made by its manufacturer, is not guaranteed or endorsed by the publisher.

## Glossary

**Table:**

ASOs	antisense oligonucleotides
AICD	activation-induced cell death
ANRIL	antisense noncoding RNA in the INK4 locus
BANCR	BRAF-activated noncoding RNA
CSCs	cancer stem cells
CTR1	Copper transporter 1
EGFR	epidermal growth factor receptor
EZH2	Enhancer of zeste homolog 2
EMT	epithelial-mesenchymal transition
GADD45A	growth arrest and DNA-damage-inducible, alpha
HOTTIP	HOXA transcript at the distal tip
IGFBP4-1	insulin-like growth factor binding protein 4-1
lncRNA	long noncoding RNA
LAD	lung adenocarcinoma
LCAL1	lung cancer-associated lncRNA-1
LSD1	Lysine-specific demethylase 1
LATS2	Large Tumor Suppressor Kinase 2
KLF2	Kruppel-like factor 2
MEG3	maternally expressed gene 3
MIR22HG	miR-22 host gene
MALAT-1	Metastasis-Associated-in-Lung-Adenocarcinoma-Transcript-1
NSCLC	non-small cell lung cancer
PCGEM1	prostate cancer gene expression marker 1
PANDAR	promoter of CDKN1A antisense DNA damage activated RNA
PNUTS	Phosphatase 1 Nuclear Targeting Subunit
PRC2	polycomb repressive complex 2
PVT1	Plasmacytoma variant translocation 1
PDE2A	phosphodiesterase 2A
RNA-seq	RNA sequencing
SCLC	small cell lung cancer
SCC	squamous cell carcinoma
TBILA	TGFβ-induced lncRNA
TUG1	taurine-upregulated gene 1
TARID	TCF21 antisense RNA inducing demethylation
XIST	X inactivate-specific transcript
ZEB1	zinc-finger E-box binding homeobox 1
